# The Role of Gender in Preparedness and Response Behaviors towards Flood Risk in Serbia

**DOI:** 10.3390/ijerph15122761

**Published:** 2018-12-06

**Authors:** Vladimir M. Cvetković, Giulia Roder, Adem Öcal, Paolo Tarolli, Slavoljub Dragićević

**Affiliations:** 1 Faculty of Security Studies, University of Belgrade, Gospodara Vučića 50, 11040 Belgrade, Serbia; vmc@fb.bg.ac.rs; 2 Department of Land, Environment, Agriculture and Forestry, University of Padova, Agripolis, viale dell’ Università 16, 35020 Legnaro, Italy; paolo.tarolli@unipd.it; 3 Independent Researcher, 06500 Ankara, Turkey; ocadem@gmail.com; 4 Faculty of Geography, University of Belgrade, Studentski Trg 3/III, 11000 Belgrade, Serbia; sasa@gef.bg.ac.rs

**Keywords:** flood risk, perception, preparedness, gender, Serbia

## Abstract

Adverse outcomes from 2014 flooding in Serbia indicated problematic response phase management accentuated by a gender imbalance. For this reason, we investigated the risk perceptions and preparedness of women and men regarding these types of events in Serbia. Face-to-face interviews, administered to 2500 participants, were conducted across 19 of 191 municipalities. In light of the current findings, men seemed to be more confident in their abilities to cope with flooding, perceiving greater individual and household preparedness. By contrast, women displayed a deeper understanding of these events. Perhaps owing to a deeper level of understanding, women demonstrated more household-caring attitudes and behaviors and were more prone to report a willingness to help flood victims at reception centers. Emergency management agencies and land planners should account for these differences in gender awareness and preparedness. Based on these findings, doing so may increase citizen participation and shared responsibility under flood hazard scenarios.

## 1. Introduction

Gender disparities exert powerful differences within societies worldwide, even in the field of disasters. Women and men are not merely at risk because of their location in time and place [1] but because of a complex mix of influential factors that include “differentiated roles and responsibilities, skills and capabilities, vulnerabilities, social relations, institutional structures, and long-standing traditions and attitudes” [2]. These social forces are thought to shape different behavioral tendencies, including those related to the ability to anticipate, prepare for, respond to, and recover from disasters [3]. The interest and recognition of different attitudes and behaviors among men and women in the wake of environmental problems have origins in the 1990s [4,5,6,7]. However, the assessment of gender differences at all levels of the disaster cycle has historically been less than satisfactory. The social research on disasters has often been approached from a mostly gender-blind perspective, mindful of some basic findings reported in the literature for years (e.g., women are more at risk for psychosocial reactions [8]). According to the gender glossary, gender blindness is “the failure to recognize that gender is an essential determinant of social outcomes impacting on projects and policies”. This social–cognitive bias can influence disaster management actions in place, affecting both physical actions and psychosocial preparedness and response [9].

As stated above, increased interest in gender inclusion in the disaster context occurred during the International Decade for Natural Disaster Reduction (1990–1999); however gender-specific guidelines were missing. In 2000, the special session of the UN General Assembly, “Gender equality, development and peace for the twenty-first century” highlighted the inefficiencies and inadequacies of existing approaches in responding to disasters [10]. Thus, the need for explicitly incorporating considerations on gender into disaster prevention, mitigation, and recovery strategies has been increasingly emphasized. The latest Sendai Framework for Disaster Risk Reduction signed off by 187 Member States on 18 March 2015 in Japan, and lead-up discussions and platforms discussed this topic more assiduously, which included a request to increase the empowerment and participation of women (and youth) [11]. The rising importance of gender-sensitive research was underlined by the United Nations Office for Disaster Risk Reduction, defined as one of the objectives for the period 2015–2020.

As a consequence, one can conclude that during the last century, progressive change regarding this issue increasingly became a current concern for the disaster management community. One emphasis is the effort devoted to understanding social power relations regarding disaster and emergency management. In particular, as promoted through UN processes, mainstreaming the role of gender includes an increased focus on integrating women in disaster policy, practice, and research.

In countries like Serbia, the context for the current study, analyzing gender differences is of particular importance. For example, during a major 2014 flood event, women were found to be particularly affected as they were weakly represented in the flood-planning response and overall decision-making processes [12]. For example, women argued that information did not reach them adequately, thus exposing gaps in risk communication. Many women’s organizations responded immediately and offered assistance in the most vulnerable areas. However, the public perception and media promulgated the idea that men were the first to react. In a policy context, the importance of protection and rescue from floods from a gender perspective has also been recognized by the Council for Gender Equality Government of the Republic of Serbia. In fact, on 29 May 2014, the council held an extraordinary meeting dedicated to this specific issue, concluding that developing more gender-sensitive statistics, indicators of vulnerability, reconstruction, and recovery was a necessary initial step. Thus, for both research- and policy-driven reasons, we conducted extensive interviews to explore and underline the differences in risk perception, preparedness, and response behaviors of both women and men in the wake of flood events in Serbia to foster increasingly targeted solutions for disaster planning and management.

### 1.1. Literature Review

Research into risk perception aims to understand the cognitive and emotional processes and behavioral choices of individuals or groups in risk-related contexts [13]. Behaviors that people enact before, during, and after disasters are the first and most critical support for the management of emergency circumstances and can contribute to the minimization of adverse effects. According to Slovic et al. [14] and Griffin et al. [15], risk encompasses an affective component responsible for two different perceptions: risk as a feeling and as a rational conception. Thus, risk is not only a combination of perceived probability but also and the feeling of worry related to it [16]. According to Cutter et al. [17], “as men and women view the world differently, it follows that they also perceive risks differently”. Gender differences may be linked to different cultural and societal contexts and dynamics. In this regard, being able to spend time watching TV or fraternizing in community centers to access hazard information [18] as well as work in the tobacco fields in Indonesia [19] gave men a greater awareness to promote responsive behavior. Female counterparts, confined to child-care and housekeeping responsibilities, as well as those watching less television and therefore receiving less knowledge of such events as a result of less interaction outside the domestic sphere, were put in jeopardy at the onset of a hazard occurrence [18,19,20]. By contrast, during Hurricane Andrew, women were largely responsible for preparing family members and expressed a higher knowledge of stocking provisions and getting the household ready for the hurricane [21]. Similar findings have been highlighted by De Marchi et al. [22]. Such findings point to emergency management messaging that understands more localized contexts and can capitalize on household tendencies, leveraging the respective gender-specific tendencies to be able to complement each other more effectively. The form of concern expressed so far can be related to the perceived likelihood of occurrence with personal consequences [23], which could affect the psychological balance [24]. Accordingly, in a recent work on Icelandic volcanic hazards regarding air travel [25], the perceptions of the necessity of restrictions were positively associated with the perceived risk of an accident and were lower for those who were more concerned about the consequences of false alarms or who had personally experienced travel disruption; they were also higher for women than men. O’Neill et al. found females to be more worried about potential flooding, and regarding perceived exposure to flood risk, gender was not found to be statistically significant [16].

Before a disaster, research shows that many individuals perceive their own risk as sufficiently low, reflecting an ‘it will not happen to me’ set of beliefs. As a result, people do not feel the need to invest voluntarily in protective measures such as strengthening their house or buying insurance [26]. Risk perceptions differently drive the willingness to protect and take action before a disaster occurs. Risk awareness is not merely a perception of hazard occurrence or the feeling of threat at the individual or household level, but also a ‘behavioral tendency’. Thus, it is related to the interest and intentions to manage more or less intensively a hazard situation. Several researchers have reported men’s higher confidence in their proactive behaviors during an emergency, rating their level of self-preparedness as significantly high [24,27,28,29]. This behavior may at least in part be driven by the social role that men usually play within the family context. By contrast, among the Wujie indigenous people interviewed in Taiwan, it was statistically determined that women felt more prepared than men when thinking about possible future hazards [30]. Similarly, women undertook more hazard adjustments than men in a US study [31]. Among these actions, there is a range of essential amenities and supplies that are helpful for the first period in a post-disaster phase. Baker [32] found that being prepared meant having additional items in the house that were essential, particularly in the response and early recovery process. According to the results obtained by Able and Nelson [33], men may see themselves as responsible for some of the necessary supplies that are needed to survive in the wake of disasters. However, in the USA, less than half of the individuals reported having a household emergency plan, including household instructions for safer locations in the case of an emergency [34].

Regarding one aspect of planning, the few studies determining gender differences have found that women are more likely to evacuate than men, perhaps because of socially constructed gender roles and dynamics [35]. Enarson [36] found that mothers rarely resisted evacuation orders, treating them more seriously than men, who may be more likely to disregard such orders. Delegating trust to local authorities and their preventive actions could magnify or shrink the response of a community by displaying a high willingness to adopt protection measures [37] in the first case, or demonstrating dramatically low preparedness in the second [30,38,39].

Official rescue attempts are made by emergency rescue services and, inevitably, those contained within relevant government authorities, supplemented by volunteer organizations [40,41]. Provision of more informal forms of voluntary assistance depends on the social order, personal characteristics, attitudes, and situational variables [42]. Assisting during and after a natural disaster can significantly contribute to reducing the consequences of disasters. Proper assistance, coming from an informed public, can create an environment where the management of a disaster event is more likely to be successful [43]. Every individual has the right to be informed of the potential risks and preparedness measures that can implement to enable secure and efficient actions [44]. Results suggest that providing specific information on how preparedness measures can be realized may increase the confidence of women in their ability to protect their property [45,46].

Thus, the findings to date support the idea that gender roles within the household and community may have direct implications for the successful prevention, mitigation, and management of hazard situations. Moderating social and demographic factors such as age, education, income, and marital status can also play a role gender-wise. As a simple example, one’s economic status enables a better absorption and recovery from losses. Perhaps less obvious is the finding that women have been found to have a higher sensitivity to possible monetary losses [47,48]. However, having adequate resources does not on its own ensure that women are not exposed to stress, anxiety, and concern about evacuation and losses, job security, or the health, safety, and well-being of family and friends [49]. Poorly maintained and inadequate infrastructures are typical of low-income women. At particular risk are single-mothers, who also tend to have lower economic means and educational levels [50,51]. Single parents typically have the same worries as two-parent households but have added responsibilities for protecting and preparing the family for an emergency. This solo status can be easily unnoticed in the recovery process [52]. Two-parent families are typically better placed, both financially and psychologically. However, even in these types of families, there can be considerable gender disparity. For example, in Kenya, 29% of the women interviewed had no formal education and 77.5% depending on their husband’s income to survive [53]. On the other hand, living in a community with a considerable proportion of highly educated women increases personal disaster preparedness and provides easier access to and seeking out of disaster-related information [54].

### 1.2. Flood Risk in Serbia

**Figure 1:**
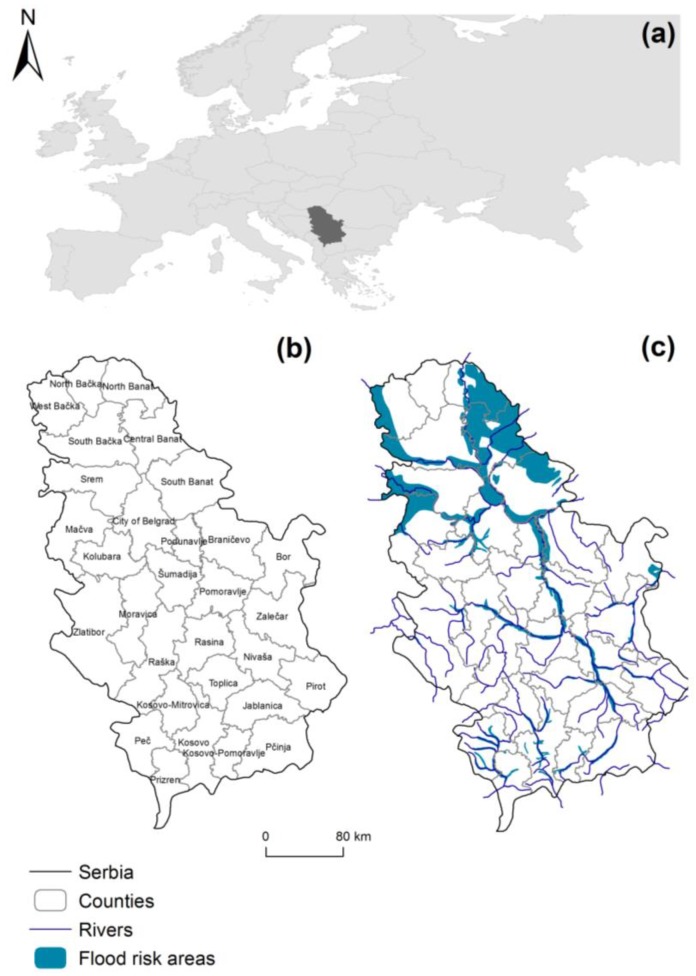
(**a**) Location of Serbia; (**b**) counties of Serbia; (**c**) flood prone areas with 100-year return period.

Floods are the most common natural hazard risks in Serbia [55,56]. Their frequency, intensity, and location across the territory make them a continuous threat to the ecological equilibrium and the social and economic development [57]. The potentially floodable area, considered for a return period of 100 years, covers an area of 16,000 km^2^ (Figure 1), where 500 large settlements, 515 industrial assets, 680 km of railroads and approximately 4000 km of roads are at risk [58].

The most vulnerable area is the northern part of Serbia, in the main river catchment of the country where the Danube River is located. The degree of vulnerability of the population and its properties are not uniform, but vary depending on the type of natural disaster and expected potential damage [55,59].

**Figure 2:**
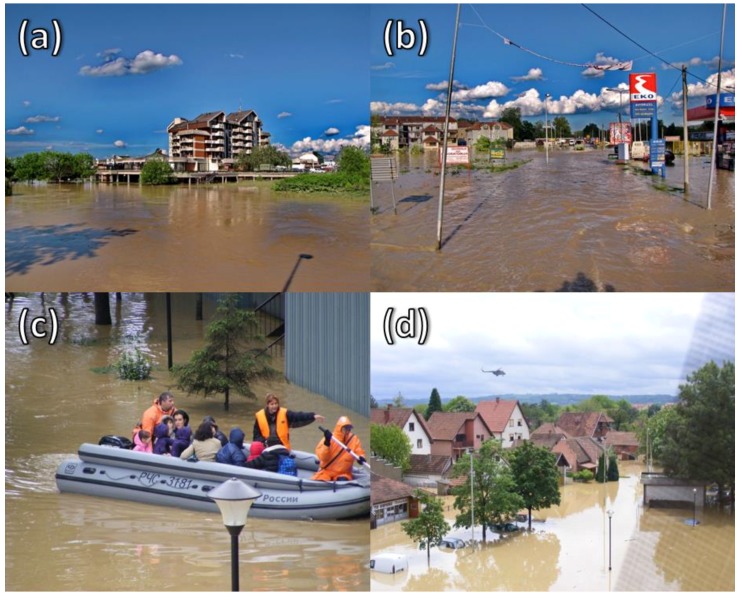
The most critical flood event occurred in 2014 in Serbia. (**a**) flooded hotel in Obrenovac; (**b**) flooded streets in Obrenovac; (**c**) citizen evacuation in Obrenovac; (**d**) rescue helicopter in Obrenovac.

In the period from 1915 to 2013, 848 flood events accounting for 133 deaths were registered [58]. In detail, the events with the most impact occurred in the Kolubara (June 1996; May 2011), Great Morava (July 1999), Kolubara and Drina (June 2001), South Morava (November 2007), West Morava, Drina and Lim (November 2009), Great Timok (February 2010), Pèinja (May 2010), and Drina (December 2010) watersheds [57]. The most critical event occurred in May 2014 (Figure 2), affecting the territories of Serbia, Bosnia-Herzegovina, and Croatia [60]. The precipitation exceeded 200 mm in a day and was the most dramatic event registered since 1961 [61], affecting more than 1.6 million people (22% of the country’s population) [60]. However, the evacuation procedures were difficult to manage because of the failure of people to move away from hazard areas. Such reactions highlighted a general mistrust of individuals in localities within the government’s actions, including evacuations, coupled with low levels of awareness and preparedness [62,63,64].

## 2. Materials and Methods

### 2.1. Study Area

A series of 2500 face-to-face interviews were conducted during the whole of 2015 in 19 of the 191 municipalities in the Republic of Serbia (Table 1). All subjects gave their informed consent for inclusion before they participated in the study, and the University of Belgrade provided ethical approval for this research. These communities were chosen for their different demographic and social characteristics, being a census-based representation of the whole population of Serbia. In these municipalities, participants were selected randomly (first stage: parts of the community in the research were selected; second stage: streets or sections of streets were determined by the level of primary causal units; third stage: each research core was determined as the path with specified start and end points of movement; fourth stage: selection of respondents was conducted following the procedure of the next birthday for adult members), with the number of respondents being proportional to its size, using a representative sampling approach [65]. Interviews were carried out for six months, and the participation rate was 96%.

### 2.2. Demographic Characteristics

The interviewees, 50.2% (1256) women and 49.8% (1244) men (Table 2), were representative of the gendered stratification of the country, which registers 51.3% women and 48.7% men [66]. The average age of respondents was 40 years old, and the most represented category was people younger than age 30 (711; 28.4%). From the sample, it appears that the majority (41.7%) had a secondary (four years) educational degree, and very few completed a higher level of education (university, 20.2%; doctoral studies, 0.4%). In the household sample, married people accounted for 54.6%.

### 2.3. Questionnaire Design

The structured questionnaire was developed using close-ended, multiple choice questions, and 5-point Likert scale questions. The first part of the questionnaire included the socio-demographic characteristics of the interviewees to assess the social background of the respondents and their gender. Subsequent sections included questions related to risk awareness, preparedness, rescue management and assistance, information and education (Appendix A, Table A1). Several published survey approaches were consulted [67,68,69,70] and adapted according to the social and flood hazard environment of Serbia. During July 2014, a pilot pre-test of the questionnaire was conducted in Batočina (central Serbia) with 75 people with the aim of checking the comprehensibility and performance of the questionnaire. In that pilot effort, participants were chosen randomly across the municipality and were interviewed in a central location (i.e., the main public square).

### 2.4. Analyses

Statistical analyses of data were conducted using the Statistical Package for the Social Sciences (SPSS) program (SPSS 20, IBM, Armonk, NY, United States of America ). First, we tested the variable ‘gender’ to validate our central hypothesis by using a multivariate analysis. A Chi-square test of independence (χ^2^) was used to determine the connection between gender and risk awareness, confidence in the positioning of household furniture, inventory of essentials, evacuation and rescue management, personal assistant of flood victims, economic support reception centers, flood occurrence information, the location of flood risk education, and the source of training. Assessment of the impact level was performed by the phi coefficient, where a higher number indicated a stronger relationship between the two variables [71]. For tables larger than 2 × 2, to assess the impact level, we used the Cramer’s V coefficient, which considers the number of degrees of freedom. To test the connection between gender and continuous dependent variables regarding individual preparedness, household preparedness, community preparedness, national preparedness, rescue management efficiency, confidence and trust, and unwillingness to get engage, a Pearson correlation was used. Before proceeding to the implementation of the statistical test, we examined the general and specific assumptions to ensure their appropriateness. The internal consistency of Likert scales for the 16 items is good with a Cronbach’s alpha of 0.80. External validity is established by using risk perception to predict intended behaviors to anticipated flooding episodes, giving χ^2^ = 33.15, *p* = 0.000.

The interaction between gender and other socio-economic or demographic variables are poorly investigated in disaster research, even if there are fundamental in programming sensitive management and assistance actions. For this reason, we gave insights regarding gender and age, income, marital status and educational level about threat appraisal, preparedness, information and communication of significant variables. We performed analysis of variance and chi-square tests of independence according to the unit of measurements of the variable implied.

## 3. Results

In order to test the central hypothesis of which gender is a predicting variable in all the stages of the disaster cycle, a multivariate regression analysis was used identifying the extent to which seven independent variables were associated with five socio-economic variables: gender, age, education, marital status, and income. According to Table 2 categories, females, young, single-headed households, university and higher educated respondents and high income people have been coded as 0; 1 has been assigned otherwise. Previous analyses showed that the assumptions of normality, linearity, multicollinearity and homogeneity of variance had not been violated. The results of the multivariate regressions (Table 3) show that the most important predictor of individual preparedness is gender (β = −0.143), and it explains 14.3% of variance, followed by the marital status (β = −0.092, 9.2%), and the level of education (β = −0.059, 5.9%). The remaining variables (e.g., age and income level) did not have significant effects on individual preparedness. This model (R^2^ = 0.032, Adj. R^2^ = 0.029, F = 15.12, *t* = 28.26, *p* = 0.000) with all mentioned independent variables explains the 29.4% variance and represent a good level of explanation based on standards in psychological research. The most important predictor of household preparedness is gender again (β = −0.049) explaining the 4.9% of variance (R^2^ = 0.009, Adj. R^2^ = 0.007, F = 4.36, *t* = 51.25. *p* = 0.000). The remaining variables did not have significant effects on household preparedness. Concerning the results of the multivariate regression with flood risk map knowledge information showed that the most important predictor is educational level (β = −0.078) explaining the 7.8% of the variance. Gender did not have significant effect in this model.

In the multivariate logistic regression model in Table 4, four independent variables (preventive measures, evacuation consent, personal assistance of flood victims, and supplies) were included. The logistic regression model related to the evacuation consent variable was statistically significant χ^2^ = 15.024 (5, *n* = 2377), *p* ≤ 0.010 and explains between 6% (Cox & Snell) and 11% (Nagelkerke) of variance. The results of the regression indicated that two predictors have a unique, statistically significant contribution to the model (income level *p* < 0.01, and education level *p* < 0.05) where income is the strongest predictor with an odds ratio of 1.44. The model including preventive measures information was statistically significant χ^2^ = 35.968 (5, *n* = 2500), *p* ≤ 0.000 and explains between 14% (Cox & Snell) and 26% (Nagelkerke) of variance. Here the results of the regression indicated that three predictors have a statistically significant contribution to the model (gender *p* < 0.05, education level *p* < 0.05 and marital status *p* < 0.01) and gender has the strongest influence with an odds ratio of 1.33. The model including information of personal assistance of flood victims was statistically significant χ^2^ = 91.84, (5, *n* = 2500), *p* ≤ 0.000 and explains between 3.6% (Cox & Snell) and 6.1% (Nagelkerke) of variance. The results of the regression indicated that two predictors have a statistically significant contribution to the model (gender *p* < 0.01, and age *p* < 0.01) and gender has the strongest influence with an odds ratio of 2.44. The model including supplies information was not statistically significant χ^2^ = 8.78 (5, *n* = 2500), *p* ≤ 0.121 but gender was the only significant predictor with an odds ratio of 1.21.

From these results we can validate our central hypothesis that gender is a predictive variable in all the stages of the disaster cycle in this research.

### 3.1. Risk Awareness

We were interested in exploring an individual’s level of threat appraisal according to a gender perspective. Threat appraisal comprises the product of objectively assessed measures of probability and consequences of risk. In this manuscript, we are evidencing the importance of all these components that construct individuals’ conceptualization of flood risk. The results showed a statistically significant difference in the awareness of a flood risk map where women (87.7%) had higher scores (*p* = 0.014). This can be explained at least in part by educational attainment. That is, women with higher levels of knowledge of flood occurrence (and maps) were also found to have a higher level of education (*p* = 0.000). Furthermore, it was found that married women and men had a higher knowledge when compared to single person families (*p* = 0.005). It appears that single headed households did not have the same concerns as those of two-headed households, perhaps grounded in the carry-over related to increased responsibilities in the family context.

However, a higher division appeared concerning the health consequences. When asking people to assess their knowledge about the health consequences of floods, it appeared that women (79.1%), compared to men (76.8%), showed more sensitivity to the health effects associated with flooding, but this was not statistically significant (Table 5). This could be ascribed to a combination of gender effects and the employment status of women. At a glance, women in Serbia are housekeepers and child carers, making them more likely to be more sensitive to environmental threats. Based on a flood likelihood scenario, on a scale of 1 to 5, it was found that the flooding likelihood of occurrence during the period of one year had a mean value of 2.58 (SD 1.36) for males and 2.53 (SD 1.34) for females, whereas it was 2.84 (SD 1.38) for males and 2.85 (SD 1.37) for females based on a 5-year probabilistic approach (Table 5). Thus, across genders in this study, there was a low awareness that these events would occur in the future, and no statistical differences were found. People seemed to perceive that they are safe from the possible occurrence of these events, even if they displayed specific knowledge about floods and their negative consequences.

For this reason, we explored in more detail whether there were some drivers concerning this lack of general awareness from a gender perspective. As expected, respondents with a higher awareness of flood risks perceived a higher flood probability (within 1 year) (men $\bar{x}$ = 2.78; women $\bar{x}$ = 2.83). In this regard, a higher education has been seen to be a significant determinant (*p* = 0.021) for men for both the flood occurrence scenarios and the opposite for female respondents (*p* = 0.013) underlined women’s higher fear derived from a low education.

### 3.2. Flood Preparedness

The other main focus of this research was an attempt to predict disaster behaviors based on a future scenario. According to the transtheoretical model of change [72], preparedness behavior readiness can be thought of in terms of different ‘stages of change’: the pre-contemplation, contemplation, preparation, action, and maintenance stages. In total, 2297 people who answered the question ‘What stage of preparedness do you feel in response to a possible flood event?’, the highest percentage consisted of those who did not intend to change or did not think about changes in the next six months (the so-called pre-contemplation or ‘non-thinking’) with a value of 60.3% (Table 6). In addition, as seen in the table, males tended to report more active stages of change (preparation, action, maintenance). Expanding on these premises, the theory of planned behavior [73] clarifies that an individual’s intentions to perform a given behavior vary according to a combination of subjective norms and perceived behavioral controls unique to each individual. For this reason, it is interesting to examine how flood preparedness varies across different demographic groups. Such variations reflect the extent to which factors can shape community-driven efforts and education, supporting efforts to be prepared for and cope with flood events.

These results were also in line with the answers provided when respondents were asked to express from 1 to 5 their level of preparedness at the individual, household, community, and national levels. In fact, women expressed lower levels of self-confidence in being prepared for a flood event, with a mean value of 2.83 versus men with a mean value of 3.13 (*r* = 0.142, *p* = 0.000). The same pattern of results was found for household preparedness (2.99 for women and 3.08 for men, *p* = 0.019, *r* = 0.047), highlighting that women were not as confident in household readiness. Any gender statistical difference has been found in regard to community and national preparedness levels, and answers underlined general low scores.

Greater perceived preparedness reported by men might be borne of the fact that, typically in Serbia, they engage in service in the army during which they are trained to manage emergency situations [12]. They are perhaps leading to a more generalized perception of being more proactive and ready when situations call for participation. Cultures, where there are family marginalization and lower levels of involvement in community networks and less preferential treatment for women, may culminate with women evaluating their preparedness to be lower than that of men including when thinking about a possible hazard event reporting to be more exposed to risk. It may also be that women are just more realistic in evaluating personal and household preparedness. As a result, and with respect to a starting point for reducing gender-based differences and risks, good knowledge of the surroundings may translate into a more exceptional individual’s capacity to cope with natural hazards.

A low level of preparedness was related to lower capacity and willingness to protect (*p* = 0.001). However, men registered a higher propensity in undertaking preventive measures with a significant value of 0.004 and a higher knowledge of the security procedures for responding to a possible flood event (*p* = 0.000). By contrast, women expressed the view that they had no time for dealing with these problems (*r* = 0.040, *p* = 0.020), especially unmarried women (unmarried $\bar{x}$ = 3.38; married $\bar{x}$ = 2.63; *p* = 0.000). That is, women’s many household, child-rearing, and related responsibilities may lead to a focus that allows them less time to consider the additional responsibility of being prepared for a possible natural hazard event.

From further analyses, we found significant relationships between preparedness and education, age, marital status and income. Poorly educated men and highly educated women perceived themselves to be the most well prepared (men *p* = 0.000; women *p* = 0.025). Concerning age, both younger female and male felt that they were more equipped compared to adults and elders perceiving a higher level of preparedness as an individual and household level. We found some statistical differences between individual/household preparedness and single women vs. married women (both >than 30 years old) (Individual preparedness: *p* = 0.000; Household preparedness: *p* = 0.000). Married women and men feel to be more prepared to overcome the negative occurrences of floods at both levels. Female-headed households generally perceived themselves to be more vulnerable to floods compared with their counterpart households with both spouses. This could be explained by the fact that households with both spouses are better placed both financially and psychologically. They are therefore able to respond to flood risks in a better mental and emotional state than are their single counterparts. Wealthier men feel to be more risk takers and well equipped (*p* = 0.008), while statistics concerning women were not significant. Generally, people with higher possessions and income feel less vulnerable to the negative occurrence being able to manage the emergency. A higher awareness of flood risks correlated positively with a higher assessment of preparedness (men *p* = 0.000; women *p* = 0.000). This means that a good awareness and preparedness campaign might translate into effective flood preparedness and mitigation measures at the individual and household levels.

Actions useful in the wake of a possible flood event include the handling of utilities (electricity, gas, water). For this reason, we asked people to assess their confidence in dealing with these. It was found that men had higher rates of confidence regarding where water valves (*p* = 0.000, male 86.5%, female 73.4%), gas valves (*p* = 0.000, male 65.3%, female 42.2%) and electricity (*p* = 0.000, male 87.8%, female 69.9%) devices were located in the household (Table 7).

We first asked people whether they stored essential amenities and, second, to choose from a list of which ones they had. More men than women reported having an inventory of essentials useful for the response process (27% and 23%, respectively). It was found that more men (a higher percentage) than women possessed a radio-transistor (*p* = 0.044), shovel (*p* = 0.000), hack (*p* = 0.000), and hoe or spade (*p* = 0.003) (Table 7). On the other hand, a higher percentage of women, compared to men, reported having water storage (*p* = 0.016) and a higher proportion of food supplies (male 59.8%, female 65.3%). In our results, women were also found to be more sensitive to essential household content protection, including significantly more women compared to men reporting that they had secured copies of important personal, financial, and insurance documents in a safe place (*p* = 0.003, female 31.4%; male 24.2%).

### 3.3. Evacuation and Rescue Management

First responders, of course, play an important role in the protection and rescue of people in disasters caused by flooding. When examining the perception of citizens regarding the efficiency of first responder actions on a scale from 1 to 5, it was found that men displayed slightly greater levels of confidence in planned emergency activities of authorities in almost all the choices provided (efficiency of fire department: men (3.56), women (3.44), *r* = −0.045; emergency medical services: men (3.55), women (3.43), *r* = −0.44; headquarters for disasters: men (3.75), women (3.69), *r* = 0.790 (Table 8).

Generally speaking, and in contrast to the standpoint of the answers given, the trust in family members as actors from whom help could be expected was significantly more pronounced for women compared to men, with mean values of 4.31 and 4.20, respectively (*r* = 0.042, *p* = 0.037). Some slight gender differences were also found regarding confidence and trust in different organizations. Females tended to rely more on police activities (*r* = 0.041, *p* = 0.043, male $\bar{x}$ = 3.25, female $\bar{x}$ = 3.36); international humanitarian organizations (*p* = 0.043, male $\bar{x}$ = 2.46, female $\bar{x}$ = 2.50), non-governmental humanitarian agencies (male $\bar{x}$ = 2.30, female $\bar{x}$ = 2.34), neighbors (male $\bar{x}$ = 3.56, female $\bar{x}$ = 3.63), religious community affiliations (*r* = 0.064, *p* = 0.002, male $\bar{x}$ = 2.38, female $\bar{x}$ = 2.43), and the army (male $\bar{x}$ = 3.56, female $\bar{x}$ = 3.58). Males, in reverse, tended towards a higher confidence in the fire department (male $\bar{x}$ = 3.63, female $\bar{x}$ = 3.61), emergency aid bodies (male $\bar{x}$ = 3.48, female $\bar{x}$ = 3.40), and themselves (male $\bar{x}$ = 3.14, female $\bar{x}$ = 3.06). Similar results were found in a study regarding trust in Serbia, which observed that the church, the police, and the army were the three principal organs in which people trusted the most for crisis management (Table 8) [66].

In contrast to the literature, in this research, it was found that men (52.6%) reported being more willing to accept evacuation orders compared to women (47.4%) (*p =* 0.023) and especially low income men (*p =* 0.002). An examination of the preferred evacuation strategies revealed statistically significant differences across all items. Men preferred to remain in the house, but moving to higher floors (male 52.6%, female 39.9%) or go to friends’ places (male 39.9%, female 32.2%). It seems that men have less confidence in the escape routes of public authorities even if they accepted evacuate orders. They expressed a preference for remaining in their houses or going to people located close by. On the other hand, women would evacuate to neighbors (female 52.6%, male 9.4%), to designated reception centers (female 16%, male 10.7%), and other empty/safer apartments (female 3.7%, male 2.9%) (*p =* 0.000) (Table 8).

Keeping oral or written response plans can also significantly contribute to more efficient evacuation strategies at the household level. For this reason, we asked people to state whether they had a plan for possible evacuation or whether they had ever held planning discussions in the case of an upcoming flood event. Although generally low rates of the possession of written plans were reported, women (4%) displayed a slightly higher percentage compared to men (3.5%) (*p* = 0.005). Additionally, females (55.2%), when compared to males (51.5%), displayed higher sensitivity to the evacuation procedures of the elderly, the disabled, and infants. There was no statistically significant difference between men and women regarding planning discussions in the case of an upcoming flood event. Here again, women demonstrated more sensitivity in taking care of aspects of household organization and safety.

### 3.4. Assistance

Gender was also evaluated as a variable predicting the willingness to assist, with men more likely to report being a volunteer during a disaster compared to women. Men (23.5%), compared to women (11.1%), reported greater assistance of flood victims, as well as higher participation with respect to economic support (men 28.1%, women 6.1%; *p* = 0.004). This latter form of assistance can be considered as ‘passive engagement’ (Table 9). A more active participation was detected from the women’s side; however, generally low base rates were reported, indicating that women (6.1%) demonstrated significantly more proactive attitudes about effective assistance at reception centers compared to men (3.7%) (*p* = 0.000).

Young women (*p* = 0.008) and men (*p* = 0.032) consider more to be engaged in assistantship to flood victims also providing economic support. The same results have been found for married individuals concerning both genders. To recall previous results, it seems that the lack of time and the higher vulnerability of single individual families make them less interested in getting engaged in the recovery process. Unexpectedly, low income women (*p* = 0.000) and men (*p* = 0.000) are more prone to support financially flood victims. This behavior is translated might be seen as ‘unquestioning obedience’ or ‘altruism’ that often comes from less wealthy individuals.

### 3.5. Information and Education

When people were asked to state the source from which they received information on floods, a gender-based relationship emerged (Table 10). Women stated to be informed by technological sources (television, press, and the Internet) and family; while their male counterparts, reported to rely more on neighbors, friends, and the place of work for their information. This might be explained by the fact that women are typically confined in the house, for their work and child rearing, making them isolated from various sources of communication, except other family members. 

By contrast, men may have more opportunities through greater interaction with the community. Furthermore, 38% of both women and men expressed a strong desire to get trained on the correct response actions in the wake of a possible flood event through the Internet or TV. Internet was the most preferred by married individuals, young people and low income respondents. Low educated (*p* = 0.000) and young men (*p* = 0.000) stated to prefer to get trained by videogames. For this reason, we asked people to assess whether they had received education on these hazards by mentioning the sources involved. Among school, family, and work, people seemed to consider family as the main source of education for floods, with a higher and statistically significant percentage of women with respect to men (*p* = 0.000). On the other hand, men (36.5%), with a higher percentage than women (28.7%), noted that their place of work was the main source of education about flood hazards (*p* = 0.000). Education from school received lower rates of endorsement among both men and women (26.5%) (Table 10).

## 4. Discussion

This research suggests that in Serbia, there may be gender differentiation across phases of the disaster cycle. However, it is important to note that it is not just a matter of difference, as underlined by Gustafson [74]. Gender is not a merely a variable that assesses the differences between men and women in the wake of disasters. It is also how living conditions, demographic and economic attributes, behaviors and beliefs reflect gender power relations in this context. Once recognized, rather than expose problems exclusively, disasters and disaster preparedness can also be seen as opportunities to facilitate or provide openings for the empowerment of traditionally marginalized groups [75].

Assessing gender discrepancies can help policy makers recognize local capacities and provide prospects for the less powerful to make disaster preparedness and relief more effective. The failure in understanding local relationships and social networks may disadvantage communities including women, men and their families and networks who face these events. One pathway for learning and integrating gender in emergency management practices includes successful stories. For example, in Bangladesh, with the introduction of improved gender-responsive disaster management, Cyclone Sidr in 2007 took a lower number of female lives when compared to previous disasters and before this policy [76]. Similar records have been found in response to tropical cyclones in Vanuatu in the South Pacific Ocean. In his report for Care International, Webb [77] demonstrated that gender-sensitive disaster risk reduction (DRR) programming contributed to reducing the impact and damage from Cyclone Pam when compared to a community that did not undertake the same plan. In Macedonia, the UNDP [78] included and trained women at the National Crisis Management Center for earthquake and flood preparedness. This initiative, undertaken in 2008, served as a best practice for gender inclusion in DRR that led to the drafting of gender-sensitive risk management plans at a national level. Successful community-based management actions depend on how public authorities’ mainstream the preparedness and recovery of men and women after disaster events, and how well gender-different realities are noticed and dealt with. Thus, to assist public authorities to organize gender-sensitive management plans, it is necessary to know how people differentially prepare and react to catastrophic events from a gender perspective.

Gender dynamics in the disaster context should be of interest to government, non-governmental, and international organizations and projects and not only at policy levels. They should also be a priority for researchers and emergency management practitioners, who need to contribute more in their studies and their practice to find a gender differentiation in how men and women perceive, prepare for, tolerate, and react to natural disasters, including in combination with different socio-cultural and economic backgrounds.

## 5. Conclusions

In this work, gender differences were found in a large sample in Serbia regarding a range of flood preparedness indicators. Although there were some variables that indicated no significant or slight differences, larger magnitude and significant differences appeared to revolve around men’s perceptions of being more prepared and being more active or willing to be involved in or led by community-level activities. Women generally reported being less confident, but perhaps had more realistic views about being prepared while also reporting more household- and family-level cares, concerns, and preparedness behaviors in selected areas. Such a pattern may be underpinned, at least to some extent, by gender-specific roles linked to the household and to community access, leading to a state of affairs that lead to less ability to connect with active social networks within the community, coupled with being less informed and able to be involved in larger decision-making processes. For this purpose, planners might consider how this may affect the way authorities can reach those people with hazard information and emergency warnings. Importantly, based on current findings coupled with other research on different gender profiles, both women and men should be seen as valuable resources that might combine complementary strengths to maximize preparedness, response, and recovery. That is, promoting more gender-related dialogue that aims to leverage the respective strengths of women and men and requires women to be increasingly empowered to take leading roles in building disaster resilience. In this work, females reported greater organization of essential supplies and emergency amenities, saving important documents, and dealing with the financial matters of the household. This should be taken not only as an advantage, but also perhaps as a proxy for a more embedded sense of prioritizing the security of the household, which makes them more motivated for arranging household and family concerns. This includes emphasizing their role in emergency management messaging for preparing the family for a possible hazard situation. Men appeared to be more confident in managing an emergency situation, including the perception that they were better prepared to take action, including physical preparedness and response. Additionally, women had fewer opportunities to maintain a high level of social networking in the community, which may lead to them being less informed. This might then underpin women expressing TV as the main channel of flood hazard information and education. Regarding the main outcomes of this research, at a political level, it is thus important to:

- Learn more about and emphasize the role of women and men in emergency management planning and messaging;
- Engage in more in-depth research on gender roles, including more in-depth qualitative or mixed methods research that uses interviewing and/or focus group methodologies on gathering more in-depth information;
- Develop strategies to empower women, educate men, and promote the genders working together synergistically to prepare effectively while also perhaps, at the same time, overcoming gender stereotypes;
- Promote gender-sensitive preparedness by using networks that appeal to and advocate for women, including those that have a long history of assessing and addressing public health issues (e.g., women’s social and health care providers);
- Use a range of communication channels for increasing hazard knowledge and preparedness, including gender-related scenarios or case studies that appeal to people and promote empowerment and working cooperatively together within households and communities;
- Include flood hazard education in children’s school curricula (e.g. education on gender empowerment and cooperation in the context of creating a current and future population that has resilience and risk management knowledge and skills) with the purpose to prepare for and solve problems linked to a range of risk scenarios in life such as flooding and other natural hazards.

Based on the current quantitative research, there is an increasing need for more gender-focused mixed methods research to contextualize gender discrepancies in more depth and at a local scale. Doing so can better target and tailor disaster management planning and preparedness, response, and recovery education campaigns. Such work could result, perhaps even quite significantly, in fewer victims of events such as floods, lessening economic losses, and reducing other consequences.

## Figures and Tables

**Table 1:** Name and ID code of the municipalities involved in the survey. The number of interviews is also shown. The location of the municipalities involved and the complete table of all Serbian municipalities (with ID) are shown in the Supplementary Material (Figure S1; Table S1).

ID	Municipality	Interviews
19	Kraljevo	141
27	Šabac	140
34	Novi Sad	150
47	Obrenovac	178
57	Kragujevac	191
60	Smederevska Palanka	205
70	Smederevo	145
100	Rekovac	50
102	Kruševac	180
115	Paraćin	147
125	Batočina	80
126	Lapovo	39
128	Svilajnac	115
147	Sremska Mitrovica	174
149	Loznica	149
151	Bajina Bašta	50
152	Užice	147
154	Priboj	122
182	Sečanj	97

**Table 2:** Basic socio-economic and demographic information of respondents (*n* = 2500) in a gendered classification. In brackets there are percentages. Missing values in the sums correspond to people that did not complete the questionnaire entirely.

Variable	Category	Total	Male	Female
Age (years)	Young (18–38)	1265 (50.6)	594 (46.96)	671 (53.04)
	Adult (39–68)	1182 (47.28)	623 (52.71)	559 (47.29)
	Old (>68)	53 (2.12)	27 (50.94)	26 (49.06)
Education level	Compulsory education ^1^	1987 (79.08)	1025 (51.58)	962 (48.42)
	University and higher ^2^	513 (20.92)	219 (42.69)	294 (57.31)
Marital status	Single-headed household ^3^	644 (45.36)	317 (49.22)	327 (50.78)
	Two-headed household ^4^	1856 (54.64)	927 (49.95)	929 (50.05)
Income ^5^	Low income	1663 (66.5)	834 (50.15)	829 (49.85)
	High income	666 (33.5)	343 (51.50)	323 (48.50)

^1^ Primary school degree (*n* = 180); Secondary degree—3 years (*n* = 520); Secondary degree—4 years (*n* = 1042); High school diploma (*n* = 245). ^2^ Bachelor degree (*n* = 439); Master degree (*n* = 65); PhD or equivalent (*n* = 9). ^3^ Single (*n* = 470); Divorced (*n* = 99); Widow/Widower (*n* = 75). ^4^ Married (*n* = 1856). ^5^ Considered below and above the national monthly average net salary. Retrieved from: http://publikacije.stat.gov.rs/G2018/PdfE/G20181260.pdf.

**Table 3:** Results of a multivariate regression analysis concerning individual and household preparedness and flood risk map knowledge (*n* = 2500).

Predictor Variable	Individual Preparedness			Household Preparedness			Flood Risk Map Knowledge		
	B	SE	β	B	SE	β	B	SE	β
Gender	−0.304	0.044	−0.143 **	−0.097	0.040	−0.049 *	−0.030	0.050	−0.012
Age	0.003	0.002	0.038	0.045	0.046	−0.021	0.214	0.058	0.077 **
Education	0.050	0.019	0.059 *	0.005	0.018	−0.007	0.212	0.056	−0.078 **
Marital status	−0.072	0.020	−0.092 **	−0.032	0.041	−0.016	−0.128	0.052	−0.051 *
Income	0.020	0.026	0.017	−0.043	0.043	−0.021	0.105	0.030	0.067 **

* *p* = 0.05. ** *p* ≤ 0.01. B: unstandardized (B) coefficients; SE: std. error; β: standardized (β) coefficients.

**Table 4:** Multivariate binary logistic regression analyses used to assess the explanatory power of four chosen predicting variables.

Predictor Variable	Preventive Measures		Evacuation Consent		Personal Assistance of Flood Victims		Supplies	
	B	SE	B	SE	B	SE	B	SE
Gender	0.287 *	0.116	0.045	0.119	0.945 **	0.116	0.197 *	0.094
Age	0.113	0.132	−0.088	0.141	0.625 **	0.140	−0.167	0.107
Education	−1.49 *	0.163	−0.329 *	0.137	−0.061	0.124	0.047	0.104
Marital status	−0.518 **	0.124	−0.123	0.123	0.096	0.114	0.129	0.097
Income	−0.229	0.123	0.371 **	0.125	0.127	0.121	−0.093	0.101

* *p* = 0.05. ** *p* ≤ 0.01. B: estimated logit coefficient; SE: std. error.

**Table 5:** Pearson correlation and Chi-square test results between risk awareness and gender. Standard deviations are shown in parentheses.

Variable	Sig. (2-Tailed)	Pearson Correlation	Men	Women
Awareness of flood probability in 1 year	0.387	−0.017	2.58 (1.36)	2.53 (1.34)
Awareness of flood probability in 5-year	0.856	0.004	2.84 (1.38)	2.85 (1.37)
Awareness on flood risk locally	0.020	0.330 *	2.78 (1.25)	2.83 (1.25)
**Variable**	**Sig. (2-Tailed)**	**χ^2^**	**Men**	**Women**
Having flood knowledge	0.167	1.90	76.8	79.1
Awareness of flood risk map	0.014 *	6.06	84.3	87.7
Awareness of health risk from flood	0.064	3.42	41.2	44.9

* Correlation is significant at the 0.05 level (2-tailed).

**Table 6:** Preparedness level from a gender perspective (*n* = 2297) based on the transtheoretical model. N stands for a number of respondents.

Preparedness Level	Description	Male		Female	
		*n*	%	*n*	%
Pre-contemplation	An individual does not intend to change or does not consider changes in the short term (in the next six months)	649	56.3	735	64.2
Contemplation	An individual is not prepared at present but intends to undertake certain activities in the next six months	144	12.5	147	12.8
Preparation	An individual has considered changing his/her behavior in the next month	141	12.2	100	8.7
Action	An individual has changed behavior in the recent past, but the changes did not come to fruition	101	8.8	75	6.6
Maintenance	An individual has changed his/her behavior, and these changes were initialized	45	3.9	37	3.2
Total:		1153	100	1144	100

**Table 7:** Pearson correlation and Chi-square test results between flood preparedness and gender. Likert scales means are shown and standard deviations are presented in parenthesis for the first two set of variables.

Category	Variable	Male	Female	Sig. (2-Tailed)	Pearson Correlation
Perception of preparedness	Individual preparedness	3.13 (1.06)	2.83 (1.01)	0.000	−0.142 **
	Household preparedness	3.08 (0.995)	2.99 (0.968)	0.019	−0.047 *
	Community preparedness	2.96 (1.16)	2.94 (1.15)	0.568	−0.012
	National preparedness	2.84 (1.10)	2.88 (1.11)	0.310	0.020
The reason for not taking precautions	Expectation from others	2.63 (1.36)	2.68 (1.29)	0.378	0.018
	Not being at risk	2.93 (1.48)	2.91 (1.41)	0.736	−0.007
	Not having time	2.57 (1.32)	2.70 (1.35)	0.020	0.047 *
	Expensive	2.74 (1.27)	2.77 (1.36)	0.638	0.010
	Fail to provide safety	2.88 (1.36)	2.91 (1.25)	0.077	0.036
	Not prevent the consequences	2.86 (1.36)	2.92 (1.35)	0.401	0.017
**Category**	**Variable**	**Male**	**Female**	**Sig. (2-Tailed)**	**χ^2^**
Inventory of essentials	Radio-transistor	19.5	15	0.044 *	4.04
	Shovel	46.6	32.9	0.000 **	24.30
	Hack	32.4	18.5	0.000 **	31.41
	Hoe, spade	37	28.9	0.003 **	9.13
	Water storage	41.3	51.7	0.016 *	8.240
	Food	59.8	65.3	0.298	0.350
Confidence in the positioning of house furniture	Water valves	86.5	73.4	0.000 **	77.85
	Gas valves	65.3	42.2	0.000 **	112.1
	Electricity	87.8	69.9	0.000 **	110.2

* Correlation is significant at the 0.05 level (2-tailed), ** Correlation is significant at the 0.01 level (2-tailed).

**Table 8:** Pearson correlation and Chi-square test results between gender and evacuation and rescue management. Likert scales means are shown, and standard deviations are presented in parenthesis for the first two set of variables.

Category	Variable	Men	Women	Sig. (2-Tailed)	Pearson Correlation
Rescue management efficiency	Police efficiency	3.30 (1.29)	3.27 (1.27)	−0.013	0.528
	Fire Department efficiency	3.56 (1.27)	3.44 (1.31)	0.021	−0.045 *
	Ambulance service efficiency	3.55 (1.17)	3.44 (1.27)	0.019	−0.44 *
	Army efficiency	3.75 (1.30)	3.69 (1.36)	0.245	−0.024
	Headquarters emergency situations efficiency	3.35 (1.32)	3.36 (1.40)	0.005	0.790 **
Confidence and trust	Family member	4.20 (1.27)	4.31 (1.18)	0.037	0.042 *
	Neighbors	3.56 (1.28)	3.63 (1.21)	0.148	0.029
	International humanitarian organization	2.39 (1.18)	2.43 (1.11)	0.419	0.016
	Non-governmental organization	2.46 (1.21)	2.50 (1.13)	0.379	0.018
	Religious community	2.31 (1.25)	2.47 (1.19)	0.002	0.064 **
	Police	3.25 (1.37)	3.36 (1.25)	0.043	0.041 *
	Fire department	3.63 (2.27)	3.61 (1.19)	0.726	−0.007
	Emergency head	3.48 (1.23)	3.40 (1.24)	0.122	−0.031
	Army	3.56 (1.36)	3.58 (1.32)	0.768	0.006
	Self-organized	3.14 (1.33)	3.06 (1.34)	0.166	−0.028
	**Variable**	**Men**	**Women**	**Sig. (2-Tailed)**	**χ^2^**
Escape route	Consent to evacuate	52.6	47.4	0.023 *	0.880
	Home—higher floors	52.6	39.9	0.000 **	22.24
	Friends’ house	39.9	32.2		
	Neighbors	9.4	52.6		
	Reception centers	10.7	16		
	Empty/Safer apartments	2.9	96.3		
Evacuation plan	Evacuation plan for vulnerable family members	3.5	4	0.005 **	−0.06
	Family dialogue on evacuation plan	16.6	14	0.117	4.28

** Correlation is significant at the 0.01 level (2-tailed), * Correlation is significant at the 0.05 level (2-tailed).

**Table 9:** Pearson correlation and Chi-square test results between assistance and gender. Likert scales mean are shown and standard deviations are presented in parenthesis for the first set of variables.

Category	Variable	Men	Women	Sig. (2-Tailed)	Pearson Correlation
Unwillingness to become engaged	Any difference	2.65 (1.24)	2.58 (1.25)	0.217	−0.026
	Expected from others	2.76 (1.21)	2.70 (1.22)	0.294	−0.22
	State body tasks	2.98 (1.21)	2.93 (1.22)	0.316	−0.021
	Expected from peers	2.98 (1.21)	2.93 (1.27)	0.041	−0.043 *
	Lack of time	2.42 (1.19)	2.29 (1.20)	0.338	−0.020
	High cost	2.65 (1.27)	2.42 (1.20)	0.007	−0.056 **
	**Variable**	**Male**	**Female**	**Sig. (2-Tailed)**	**χ^2^**
Type of assistance	Personal assistance of flood victims	23.5	11.1	0.000 **	63.6
	Economic support	28.1	33.6	0.004 **	8.38
	Reception Centers	3.7	6.1	0.000 **	6.32

** Correlation is significant at the 0.01 level (2-tailed), * Correlation is significant at the 0.05 level (2-tailed).

**Table 10:** Chi-square test results between gender and information and education-predicting variables.

Category	Variable	Male	Female	χ^2^	Sig. (2-Tailed)
Flood occurrence information	Family members	29.3	33.1	3.87	0.015 *
	Neighbors	18.3	13.8	8.46	0.049 *
	Friends	12.3	9.5	4.47	0.004 **
	Relatives	12.7	11.3	0.995	0.034 *
	School	12.8	15.4	3.23	0.319
	College	6.9	4.5	5.72	0.072
	Work	16.8	11.8	11.80	0.017 *
	Religious community	2.8	2.4	0.199	0.001 **
	Television	54.8	63	16.27	0.655
	Radio	16.3	15.2	0.403	0.000 **
	Press	29.5	33.9	5.11	0.526
	Internet	24.4	33	20.74	0.024 *
The place of flood risk education	School	36.5	28.7	2.11	0.347
	Family	41.4	44.1	4.92	0.000 **
	Work	36.5	28.7	16.88	0.000 **
Source of training	Television	62.3	62.4	0.000	1.00
	Radio	13.3	11.8	1.20	0.273
	Video games	3.1	0.5	20.11	0.000 **
	Internet	20.6	28	10.01	0.000 **
	Lecture	30.3	31.4	0.318	0.573

** Correlation is significant at the 0.01 level (2-tailed), * Correlation is significant at the 0.05 level (2-tailed).

## Supplementary Materials

The following are available online at http://www.mdpi.com/1660-4601/15/12/2761/s1, Figure S1: Study areas location (grey). Numbers refer to the ID of the municipality, Table S1: The ID of the municipality and belonging county in Serbia.

## Appendix A

**Table A1:** Set of questionnaire variables and units of measurement.

Variable	Units of Measurement
*Threat appraisal*	
Flood knowledge	Dummy variable (yes/no/not sure)
Flood risk map knowledge	Dummy variable (yes/no/not sure)
Flood-related health risks	Dummy variable (yes/no/not sure)
1-year flood likelihood scenario	5-Point Likert scale
5-year flood likelihood scenario	5-Point Likert scale
Feeling of danger	5-Point Likert scale
*Flood preparedness*	
Preparedness	Transtheoretical model (Citizens Corps 2006)
Individual preparedness	5-Point Likert scale
Household preparedness	5-Point Likert scale
Community preparedness	5-Point Likert scale
National preparedness	5-Point Likert scale
Unwillingness to protect	Multiple choice question: (1) Expectation from others, (2) Not being at risk, (3) Not having time, (4) Expensive, (5) Fail to provide safety, (6) Not prevent the consequences
Preparation usefulness for the future	5-Point Likert scale
Confidence in the positioning of house furniture	Dummy variable (yes/no): (1) Water valves, (2) Gas valves, (3) Electricity
Confidence in handling house furniture	Dummy variable (yes/no): (1) Water valves. (2) Gas valves, (3) Electricity
Inventory of essentials	Dummy variable (yes/no): (1) radio-transistor, (2) shovel, (3) hack, (4) hoe, spade, (5) water storage, (6) food
Confidence in the location of financial documents	Dummy variable (yes/no)
*Evacuation and rescue management*	
Escape route	Multiple choice question: (1) Home- Higher floors, (2) Friends’ house, (3) Neighbors, (4) Reception centers, (6) Empty/Safer apartments
Consent to evacuate	Dummy variable (yes/no)
Family dialogue on evacuation plan	Dummy variable (yes/no)
Evacuation plan for vulnerable family members	Dummy variable (yes/no)
Rescue management efficiency	5-Point Likert scale: (1) Police, (2) Fire Department, (3) Ambulance service, (4) Army, (5) Headquarters emergency situations
Confidence and trust	5-Point Likert scale: (1) Family member, (2) Neighbors, (3) International humanitarian organization, (4) Non-governmental organization, (5) Religious community, (6) Police, (7) Fire department, (8) Emergency head, (9) Army, (10) Self-organized
*Assistance*	
Willingness to assist community recovery	Dummy variable (yes/no)
Type of assistance	Dummy variable (yes/no): (1) Personal assistance of flood victims, (2) Economic support (3) Reception Centers
Unwillingness to become engaged	Level of agreement on a 5-point Likert scale: (1) Any difference, (2) Expected from others, (3) State body task, (4) Expected from peers, (5) Lack of time, (6) High cost
*Information and education*	
Flood occurrence information	Dummy variable (yes/no): (1) Family members, (2) Neighbors, (3) Friends, (4) Relatives, (5) School, (6) College, (7) Work, (8) Religious community, (9) Television, (10) TV, (11) Radio, (12) Press, (13) Internet
Flood risk education	Dummy variable (yes/no): (1) School, (2) Family, (3) Work
Desire to be trained	Dummy variable (yes/no)
Preferable training source	Dummy variable (yes/no): (1) Television, (2) Radio, (3) Video games, (4) Internet, (5) Lecture
