# Supplementary material for: The Role of Gender in Preparedness and Response Behaviors towards Flood Risk in Serbia
Source: Int J Environ Res Public Health. 2018 Dec 6;15(12):2761. doi: 10.3390/ijerph15122761 (PMC6313390; doi:10.3390/ijerph15122761)
Supplement: Supplementary file 1 [file ijerph-15-02761-s001.pdf]

International Journal of
Environmental Research and Public Health

MDPI

Supplementary material

The Role of Gender in Preparedness and Response Behaviors towards Flood Risk in Serbia

Vladimir M. Cvetković [1], Giulia Roder [2]*, Adem Öcal [3], Paolo Tarolli [2] and Slavoljub Dragićević [4]

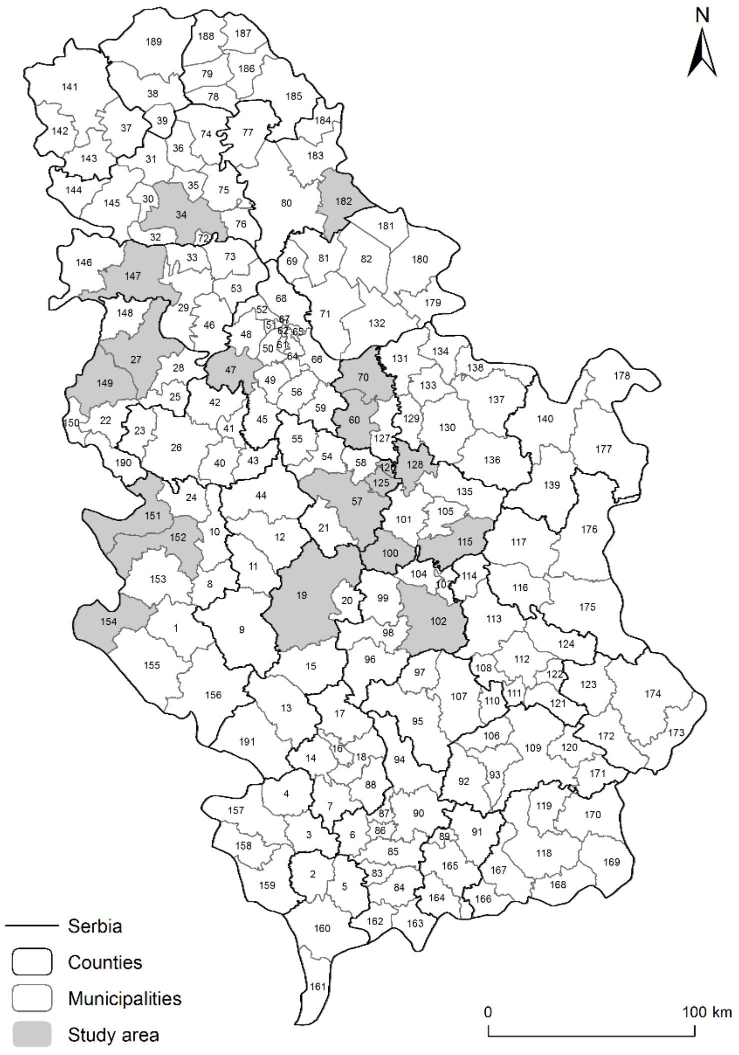

Figure S1. Study areas location (grey). Numbers refer to the ID of the municipality.

Table S1. The ID of the municipality and belonging county in Serbia.

ID	Municipality	County	ID	Municipality	County
1	Nova Varoš	Zlatibor	96	Brus	Rasina
2	Orahovac	Prizren	97	Blace	Toplica
3	Klina	Peč	98	Aleksandrovac	Rasina
4	Istok	Peč	99	Trstenik	Rasina
5	Suva Reka	Prizren	100	Rekovac	Pomoravlje
6	Glogovac	Kosovo	101	Jagodina	Pomoravlje

International Journal of
Environmental Research
and Public Health

7	Srbica	Kosovo-Mitrovica	102	Kruševac	Rasina
8	Arilje	Zlatibor	103	Ćicevac	Rasina
9	Ivanjica	Moravica	104	Varvarin	Rasina
10	Požega	Zlatibor	105	Ćuprija	Pomoravlje
11	Lucani	Moravica	106	Bojnik	Jablanica
12	Cacak	Moravica	107	Prokuplje	Toplica
13	Novi Pazar	Raška	108	Merošina	Nivaša
14	Zubin Potok	Kosovo-Mitrovica	109	Leskovac	Jablanica
15	Raška	Raška	110	Žitoradja	Toplica
16	Zvečan	Kosovo-Mitrovica	111	Doljevac	Nivaša
17	Leposavić	Kosovo-Mitrovica	112	Niš	Nivaša
18	Kosovska Mitrovica	Kosovo-Mitrovica	113	Aleksinac	Nivaša
19	Kraljevo	Raška	114	Ražanj	Nivaša
20	Vrnjačka Banja	Raška	115	Paraćin	Pomoravlje
21	Knić	Šumadija	116	Sokobanja	Zalečar
22	Krupanj	Mačva	117	Boljevac	Zalečar
23	Osecina	Kolumbara	118	Vranje	Pčinja
24	Kosjerić	Zlatibor	119	Vladičin Han	Pčinja
25	Koceljeva	Mačva	120	Vlasotince	Jablanica
26	Valjevo	Kolumbara	121	Gadzin Han	Nivaša
27	Šabac	Mačva	122	Niška Banja	Nivaša
28	Vladimirci	Mačva	123	Bela Palanka	Pirot
29	Ruma	Srem	124	Svrljig	Nivaša
30	Bački Petrovac	South Bačka	125	Batočina	Šumadija
31	Vrbas	South Bačka	126	Lapovo	Šumadija
32	Beočin	South Bačka	127	Velika Plana	Podunavlje
33	Irig	Srem	128	Svilajnac	Pomoravlje
34	Novi Sad	South Bačka	129	Žabari	Braničevo
35	Temerin	South Bačka	130	Petrovac	Braničevo
36	Srbobran	South Bačka	131	Pozarevac	Braničevo
37	Kula	West Bačka	132	Kovin	South Banat
38	Bačka Topola	North Bačka	133	Malo Crnice	Braničevo
39	Mali Ioš	North Bačka	134	Veliko Gradiste	Braničevo
40	Mionica	Kolumbara	135	Despotovac	Pomoravlje
41	Lajkovac	Kolumbara	136	Zagubica	Braničevo
42	Ub	Kolumbara	137	Kučevo	Braničevo
43	Ljig	Kolumbara	138	Golubac	Braničevo
44	Gornji Milanovac	Moravica	139	Bor	Bor
45	Lazarevac	City of Belgrade	140	Majdanpek	Bor
46	Pecinci	Srem	141	Sombor	West Bačka
47	Obrenovac	City of Belgrade	142	Apatin	West Bačka
48	Surčin	City of Belgrade	143	Odzaci	West Bačka
49	Barajevo	City of Belgrade	144	Bač	South Bačka
50	Čukarica	City of Belgrade	145	Bačka Palanka	South Bačka
51	Novi Beograd	City of Belgrade	146	Šid	Srem

52	Zemun	City of Belgrade	147	Sremska Mitrovica	Srem
53	Stara Pazova	Srem	148	Bogatic	Mačva
54	Topola	Šumadija	149	Loznica	Mačva
55	Arandjelovac	Šumadija	150	Mali Zvornik	Mačva
56	Sopot	City of Belgrade	151	Bajina Bašta	Zlatibor
57	Kragujevac	Šumadija	152	Užice	Zlatibor
58	Raca	Šumadija	153	Čajetina	Zlatibor
59	Mladenovac	City of Belgrade	154	Priboj	Zlatibor
60	Smederevska Palanka	Podunavlje	155	Prijepolje	Zlatibor
61	Rakovica	City of Belgrade	156	Sjenica	Zlatibor
62	Savski Venac	City of Belgrade	157	Peć	Peč
63	Vračar	City of Belgrade	158	Dečani	Peč
64	Voždovac	City of Belgrade	159	Djakovica	Peč
65	Zvezdara	City of Belgrade	160	Prizren	Prizren
66	Grocka	City of Belgrade	161	Gora	Prizren
67	Stari Grad	City of Belgrade	162	Strpce	Kosovo
68	Palilula	City of Belgrade	163	Kačanik	Kosovo
69	Opovo	South Banat	164	Vitina	Kosovo-Pomoravlje
70	Smederevo	Podunavlje	165	Gnjilane	Kosovo-Pomoravlje
71	Pančevo	South Banat	166	Preševo	Pčinja
72	Sremski Karlovci	South Bačka	167	Bujanovac	Pčinja
73	Indjija	Srem	168	Trgovšte	Pčinja
74	Bečej	South Bačka	169	Bosilegrad	Pčinja
75	Abalj	South Bačka	170	Surdulica	Pčinja
76	Titel	South Bačka	171	Crna Trava	Jablanica
77	Novi Bečej	Central Banat	172	Babušnica	Pirot
78	Ada	North Banat	173	Dimitorvgrad	Pirot
79	Senta	North Banat	174	Pirot	Pirot
80	Zrenjanin	Central Banat	175	Knjaževac	Zaječar
81	Kovačica	South Banat	176	Zaječar	Zaječar
82	Alibunar	South Banat	177	Negotin	Bor
83	Stimlje	Kosovo	178	Kladovo	Bor
84	Urosevac	Kosovo	179	Bela Crkva	South Banat
85	Lipljan	Kosovo	180	Vršac	South Banat
86	Kosovo Polje	Kosovo	181	Plandište	South Banat
87	Obilić	Kosovo	182	Sečanj	Central Banat
88	Vučitrn	Kosovo-Mitrovica	183	ÄitiÜte	Central Banat
89	Novo Brdo	Kosovo-Pomoravlje	184	Nova Crnja	Central Banat
90	Pristina	Kosovo	185	Kikinda	North Banat
91	Kosovska Kamenica	Kosovo-Pomoravlje	186	Čoka	North Banat
92	Medveđa	Jablanica	187	Novi Kneževac	North Banat
93	Lebane	Jablanica	188	Kanjiža	North Banat
94	Podujevo	Kosovo	189	Subotica	North Bačka
95	Kuršumlija	Toplica	190	Ljubovija	Mačva
			191	Tutin	Raška

International Journal of
*Environmental Research
and Public Health*
